# Enhancing Plant Stress Tolerance: The Role of *LcWRKY40* Gene in Drought and Alkaline Salt Resistance in Tobacco and Yeast

**DOI:** 10.3390/ijms251810149

**Published:** 2024-09-21

**Authors:** Jianan Guo, Yipeng Zhao, Huihui Cheng, Ruiqiang Yu, Baoxiang Gu, Qiuhui Wang, Jitao Zhang, Shenglin Li, Qingjie Guan

**Affiliations:** 1 Key Laboratory of Saline-Alkali Vegetation Ecology Restoration, Ministry of Education, College of Life Sciences, Northeast Forestry University, Harbin 150040, China; gjn00@nefu.edu.cn (J.G.); zypsy@nefu.edu.cn (Y.Z.); chenghh@nefu.edu.cn (H.C.); yuruiqiang@nefu.edu.cn (R.Y.); gutongxue@nefu.edu.cn (B.G.); wangqiuhui@nefu.edu.cn (Q.W.); 2 Key Laboratory of Soybean Molecular Design Breeding, Northeast Institute of Geography and Agroecology, The Chinese Academy of Sciences, Changchun 130102, China; zhangjitao@neigae.ac.cn; 3 School of Life Science and Agriculture Forestry, Qiqihar University, Qiqihar 161000, China; dr-shenglinli@qqhru.edu.cn

**Keywords:** *Leymus chinensis*, *LcWRKY40* gene, drought resistance, saline-alkaline resistance

## Abstract

*Leymus chinensis*, a halophytic perennial grass belonging to the Poaceae family, thrives in saline-alkali grasslands and harbors a rich repository of resistance-related genetic resources. This study focused on deciphering the stress-responsive mechanisms of *L. chinensis* by conducting transcriptomic sequencing under NaHCO_3_ stress, which resulted in the annotation of a segment corresponding to the 51WRKY gene. The alkali-induced gene *LcWRKY40* (QIG37591) was identified by phylogenetic analysis. Real-time quantitative PCR analysis was performed on *L. chinensis* plants subjected to PEG6000 and alkaline salt (NaHCO_3_) stress, and the results indicated that the *LcWRKY40* gene was upregulated in both the leaves and roots. The localization of the LcWRKY40 protein was confirmed by the use of green fluorescent protein (GFP) fusion technology in transformed rice protoplast cells. The GAL4-driven transformation of the *LcWRKY40* gene in INVScI yeast cells, which exhibited enhanced tolerance upon overexpression of the *LcWRKY40* gene under mannitol and alkaline salt (NaHCO_3_) stress conditions. Under drought stress using mannitol, the fresh weight of Nicotiana tabacum overexpressing the *LcWRKY40* gene was significantly higher than that of wild-type(WT) tobacco. Through drought and salt alkali stress, we found that overexpressed tobacco at different stages always outperformed the wild type in terms of fresh weight, SOD, MDA, and Fv/Fm. This study provides preliminary insights into the involvement of the *LcWRKY40* gene in responding to drought and alkaline salt stresses, highlighting its role in enhancing plant resistance to drought and saline-alkaline conditions. These findings lay the foundation for future molecular breeding strategies aimed at improving grass resistance from different aspects.

## 1. Introduction

Plants employ a self-protective regulatory mechanism when subjected to environmental stress during growth [1], often involving transcription factors that directly or indirectly modulate the expression of pertinent genes to enhance stress tolerance. *Leymus chinensis*, a predominant species in the saline-alkali grasslands of the Songnen Plain, has significant ecological and cultivation advantages in terms of ecophysiological stress and biological adaptation [2]. It belongs to the Poaceae family and is characterized by both drought resistance and tolerance to soda saline-alkali conditions, housing a rich repository of resistance-related genetic resources [3,4]. The molecular underpinnings of phenotypic variation are frequently linked to changes in gene expression, thereby impacting phenotypic variability and plant adaptive evolution [5]. Gene expression is influenced by both internal transcriptional regulatory factors and external environmental factors, yielding gene expression responses [6]. Employing RNA sequencing technology, Sun et al. performed differential gene expression analysis of *L. chinensis* in four soil types: farmland, forest, saline-alkali, and sandy soil. Kyoto Encyclopedia of Genes and Genomes (KEGG) pathway analysis revealed that secondary metabolism-related biosynthesis was associated with plant growth and stress responses. Specifically, 1345 genes whose expression was activated and 510 genes whose expression was silenced were identified in *L. chinensis* root systems within saline-alkali soil [7]; these genes included WRKY transcription factors. WRKY transcription factors play pivotal regulatory roles in plant responses to biotic and abiotic stress. Studies have demonstrated that overexpressing *ClWRKY20* in transgenic *Arabidopsis* enhances salt (NaCl) and cold tolerance [8]. Overexpression of *PtWRKY39* enhances drought and salt–alkali resistance during seed germination and vegetative growth in transgenic tobacco [9]. The *WRKY28* gene of *Mongolian poplar* enhances salt (NaCl) and alkali (NaHCO_3_) tolerance by regulating reactive oxygen species clearance pathways [10]. *VaWRKY14* overexpression in *Arabidopsis* induces drought resistance possibly by regulating stress-related gene expression levels [11]. *MdWRKY56,* an important positive regulator of drought stress tolerance in apple plants, has a positive function in regulating osmotic stress and drought stress tolerance [12]. *DcWRKY5* is a positive regulator involved in the drought and salt tolerance of the medicinal plant *Dendrobium chrysotoxum*. Overexpression of *DcWRKY5* modulates the expression of genes associated with salt and drought stress responses [13]. Glutathione (GSH) in *Arabidopsis* interacts with mitogen-activated protein kinase 3 (MPK3) and the transcription factor *WRKY40*, inducing stress responses to osmotic and cold stress and enhancing resistance through *AtWRKY40*-mediated MPK3 expression [14]. *FcWRKY70* plays a role in drought tolerance by regulating ADC (arginine decarboxylase) expression that partially promotes putrescine production [15]. Additionally, *IlWRKY70* in Iris enhances plant salt and drought tolerance by modulating ROS (reactive oxygen species) [16].

Our studies indicate that transgenic *LcWRKY40* tobacco plants exhibit varying degrees of disease resistance against pathogens, particularly displaying increased resistance to rice blast fungus and Phytophthora blight [17]. However, the transcription factors of *LcWRKY40* have not been explored in terms of drought and salt alkali resistance. Therefore, we treated *L. chinensis* plants at the fourth leaf stage with NaHCO_3_ solution and identified the upregulated *LcWRKY40* gene through transcriptomic sequencing. This study aimed to investigate the time-dependent expression patterns of the *LcWRKY40* gene under drought and alkaline salt (NaHCO_3_) stresses by subsequently constructing a pYES2 yeast overexpression vector and transforming INVScI yeast cells to analyze its regulatory role in osmotic and saline-alkali stress sensitivity. Furthermore, we generated overexpressed *Nicotiana tabacum* plants to assess their resistance during the germination and growth periods. Through examination of plant performance post-alkaline salt treatment and analysis of physiological indicators, we sought to elucidate the regulatory role of the *LcWRKY40* transcription factor in conferring drought and saline-alkali tolerance, providing a foundation for using gene editing in molecular plant breeding. 

## 2. Results

### 2.1. Cloning and Identification of LcWRKY40 Gene in Leymus chinensis

**Figure 1:**
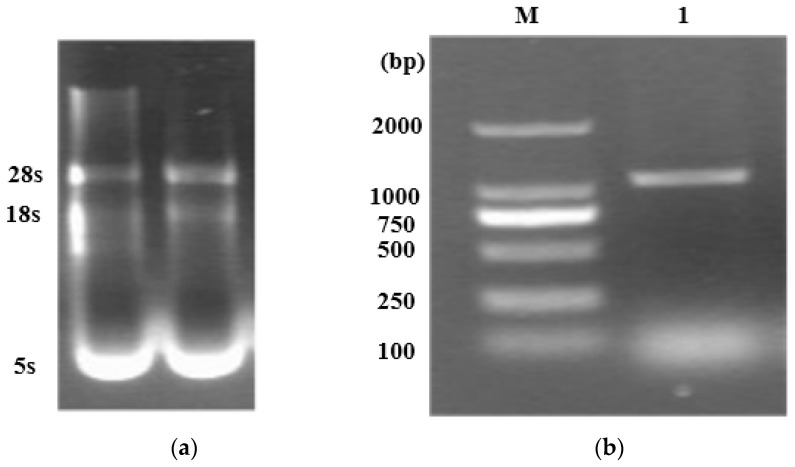
Cloning of *LcWRKY40* gene: (**a**) total RNA electrophoretic detection of sheep grass leaves; (**b**) electrophoretic detection of PCR amplification products; M: DNA standard; 1: target PCR product.

Total RNA was extracted from the leaves and roots of *L. chinensis* using a total RNA extraction kit, followed by electrophoresis analysis (Figure 1a). After reverse transcription of the total RNA from the leaves into cDNA, 1μL of the cDNA was used as a template for PCR amplification. The target band was approximately at the 1000 bp position (Figure 1b), which matches the expected size. The recovered DNA was then ligated into the pMD18-T vector, and the resulting pMD18-T-*LcWRKY40* plasmid was sequenced to confirm its correctness.

### 2.2. LcWRKY40 Gene Response to Stress over Time

**Figure 2:**
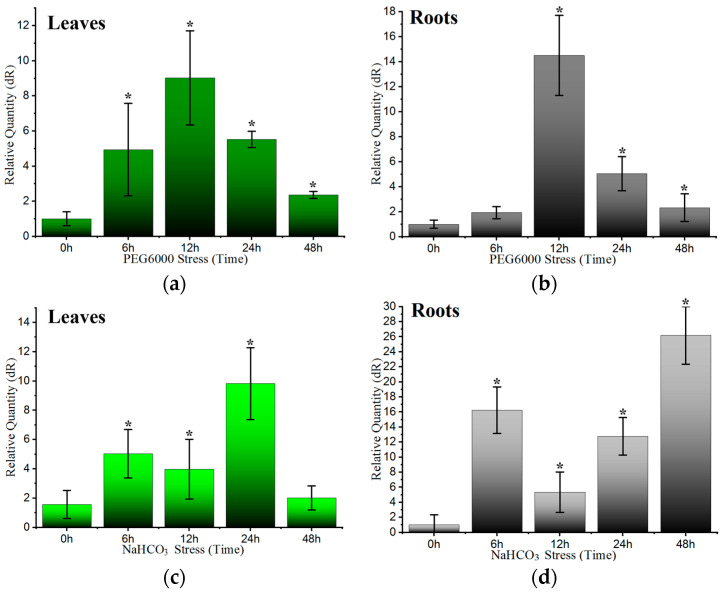
Analysis of *LcWRKY40* gene expression under drought and alkaline salt stress conditions: (**a**,**b**) analysis of *LcWRKY40* expression in the leaves and roots of *Leymus chinensis* after PEG stress, respectively; (**c**,**d**) expression of *LcWRKY40* in the leaves and roots of *Leymus chinensis* after NaHCO_3_ stress, respectively; error bars represent standard deviations of three biological replicates. * indicates significant differences (more than twofold) compared with the control.

This study employed real-time quantitative PCR to measure the expression levels of the *LcWRKY40* gene in *L. chinensis* plants subjected to drought and alkaline salt stresses. The cultivated seedlings displayed upregulated *LcWRKY40* gene expression under both the Polyethylene glycol (PEG) and NaHCO_3_ treatments. In response to PEG stress, the expression of *LcWRKY40* increased notably in the early stages, reaching its peak at 12 h post treatment, with a remarkable increase of 9.03-fold compared to that of the control at 0 h. Subsequently, the expression of these genes decreased, maintaining a 2.35-fold increase at 48 h (Figure 2a). Similarly, in the roots, exposure to PEG induced *LcWRKY40* gene expression, which reached its peak at 12 h, at 14.49-fold greater than that of the control at 0 h, followed by a decrease (Figure 2b). After NaHCO_3_ treatment, *LcWRKY40* gene expression in *L. chinensis* plants was notably upregulated, as depicted in Figure 2c,d. In both the leaves and roots, there was an overall increase in gene expression. In the leaves, exposure to NaHCO_3_ induced a peak in gene expression at 24 h, which was a 9.82-fold increase compared to that of the control at 0 h. In the roots, there was an initial increase in expression at 6 h following NaHCO_3_ treatment, followed by a decrease at 12 h. Subsequently, there was a significant increase in gene expression from 24 to 48 h in response to stress, with the highest expression observed at 48 h, reaching 26.19-fold greater than that in the control. The findings of the present study revealed increased expression of the *LcWRKY40* gene in both leaves and roots under drought and alkaline salt stresses, suggesting its involvement as a stress-responsive gene. This finding implies the potential role of the *LcWRKY40* transcription factor in modulating the adverse effects of drought and alkaline salt stresses, potentially contributing to enhanced plant resistance.

### 2.3. Protein Subcellular Localization Analysis

**Figure 3:**
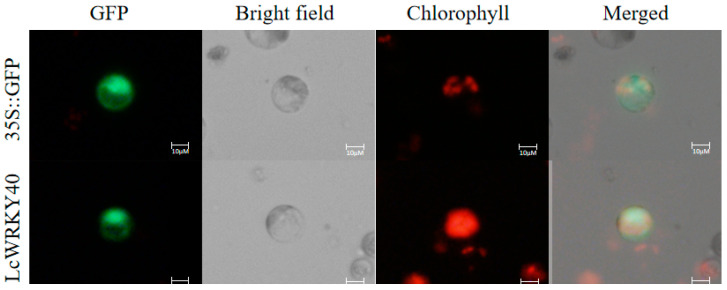
LcWRKY40 subcellular protein localization in rice protoplasts. Distribution of LcWRKY40-GFP fusion protein in rice protoplasts. Using the 35S-GFP vector as a control, Merge represents the superimposed field of GFP, bright field, and chlorophyll. The scale is 10 μm.

This study predicted the localization of the LcWRKY40 protein, which is encoded by a 350-amino-acid sequence. Analysis conducted on the https://wolfpsort.hgc.jp/ (accessed on 25 June 2024) website indicated a 13.01% probability of nuclear localization and further revealed the presence of the SRKRKS nuclear localization sequence. By employing the rice protoplast transient transformation method, the pBI121*::LcWRKY40::GFP* fusion expression vector was introduced into protoplast cells, which were subsequently cocultured for 16 h. Through laser confocal microscopy, a distinct green fluorescent protein signal was detected (Figure 3). The co-expression of the LcWRKY40 and GFP confirmed the localization of the LcWRKY40 transcription protein within the cell nucleus, which was consistent with the previous prediction. This finding suggested that the functional site of *LcWRKY40* is within the cell nucleus.

### 2.4. Overexpression of the LcWRKY40 Gene in Yeast for Drought and Salt–Alkali Tolerance

**Figure 4:**
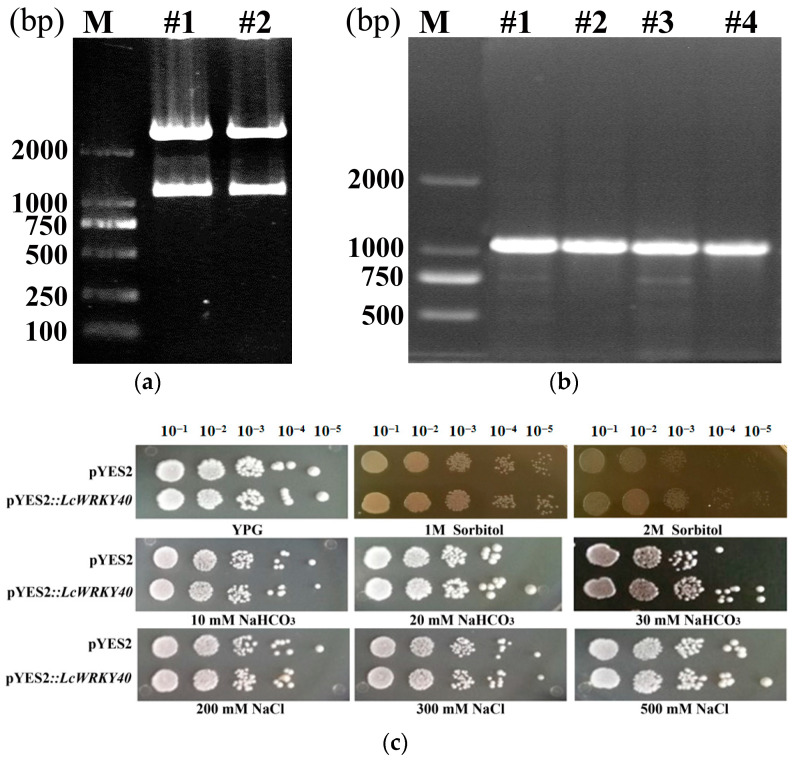
Analysis of resistance in transgenic pYES2*::LcWRKY40* yeast: (**a**) double enzyme digestion electrophoresis of pYES2*::LcWRKY40* recombinant plasmid; (**b**) PCR electrophoresis of transformed INVScI yeast clones (M is the DNA standard); (**c**) YPG for control growth medium, YPG + 1 and 2 M sorbitol media for simulated drought; 10, 20, and 30 mM NaHCO_3_ for alkaline salt stress; 200, 300, and 500 mM NaCl for salt stress.

This study involved the construction of the pYES2*::LcWRKY40* yeast expression vector for analyzing growth resistance in the INVScI strain. EcoRI and XhoI double-enzyme digestion confirmed successful construction of the pYES2*::LcWRKY40* recombinant plasmid (Figure 4a). PCR further verified the correct insertion of the construct into INVScI yeast cells (Figure 4b). Using a starting yeast concentration of OD_λ600_ = 0.6 for both pYES2*::LcWRKY40* and the pYES2 vector gene, 3 μL spots were observed on stress plates. After 72 h, consistent growth was observed on YPG solid culture medium (control). No significant difference in growth was apparent on 1 M sorbitol. However, on 2 M sorbitol, a substantial difference was observed, where the control yeast failed to grow at a 10^−4^ dilution, whereas the overexpressed *LcWRKY40* yeast clone exhibited resistance to sorbitol. The overexpressed yeast strain showed tolerance to 20 and 30 mM NaHCO_3_, although the resistance decreased as the salt concentration increased. Nevertheless, it displayed enhanced growth in the presence of alkaline salt. The yeast culture exhibited insensitivity to low-concentration NaCl stress but demonstrated growth tolerance on 500 mM NaCl (Figure 4c). The observable differences in growth between the overexpressed yeast and the control under sorbitol and alkaline salt (NaHCO_3_) stress suggested that the expression of the *LcWRKY40* gene might enhance yeast tolerance, warranting further detailed analysis and research.

### 2.5. Overexpression of the LcWRKY40 Gene in Tobacco Enhances Drought and Saline-Alkaline Tolerance

#### 2.5.1. Molecular Identification of *LcWRKY40* Overexpressed in Tobacco

**Figure 5:**
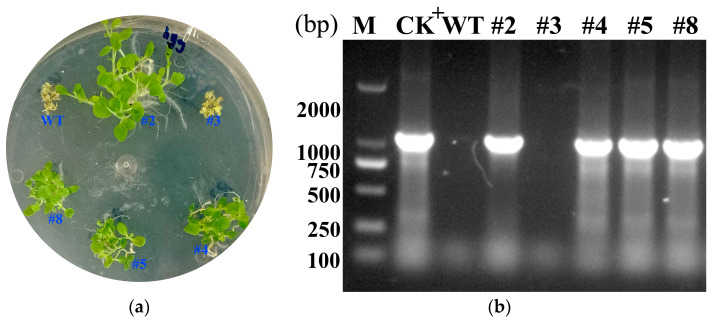
Screening for thaumatin resistance and RT-PCR detection of expression in tobacco plants transfected with the *LcWRKY40* gene: (**a**) characterization of the growth of the resistant transgenic *LcWRKY40* tobacco lines T3-#2, T3-#4, T3-#5, and #8 on 1/2 MS + 50 mg/L Hyg medium; (**b**) RT-PCR electrophoresis results for the transgenic strains; M: DNA marker; CK^+^: positive control.

To assess drought and saline-alkaline resistance in the transgenic *LcWRKY40* plants, the expression of the genes in the T3 generation was initially analyzed. Tobacco plants from T3 lines #2, #3, #4, #5, and #8 were cultivated on media supplemented with hygromycin (Hyg) (Figure 5a). The WT and line #3 failed to germinate and grow properly on hygromycin-containing media, whereas lines #2, #4, #5, and #8 exhibited germination and healthy growth. RT-PCR analysis detected nonspecific gene expression in line #3. Moreover, the leaves of lines #2, #4, #5, and #8 showed overexpression of transcripts compared to those of the WT (Figure 5b). The high expression of the *LcWRKY40* gene in these four transgenic lines (T3-#2, #4, #5, and #8) provided suitable materials for further analysis of resistance.

#### 2.5.2. Analysis of Drought Resistance in *LcWRKY40*-Overexpressing Tobacco

**Figure 6:**
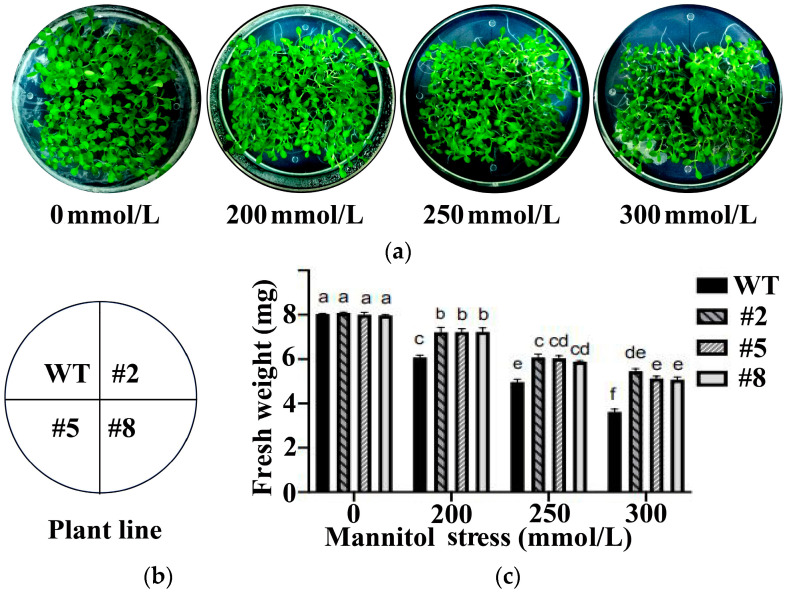
Resistance analysis of tobacco plants overexpressing the *LcWRKY40* gene during germination under mannitol stress: (**a**) wild-type and transgenic tobacco seeds grown on 1/2 MS plates supplemented with mannitol for 20 days; (**b**) is the legend of (**a**), which corresponds to WT, #2, #5, #8 of the four pictures in (**a**) each figure; (**c**) total fresh weight (mg) of 10 seedlings, and three sets of experiments were repeated; a, b, c, d, e, and f represent significant differences (*p* < 0.05).

To investigate the function of *LcWRKY40* in the stress response, plants overexpressing *LcWRKY40* were selected and bred to the T3 generation. Seedlings with consistent growth from lines #2, #5, and #8 were chosen for the mannitol stress treatment. Observations of the growth phenotypes of each plant during the mannitol stress treatment were recorded. The results are illustrated in Figure 6. Under normal growth conditions, there were no significant differences observed between the wild-type plants and the overexpression plants. However, after 20 days of mannitol stress treatment, the wild-type plants exhibited stunted growth, with some wilting of the leaves, indicating clear inhibition of growth. In contrast, the overexpression plants were taller and had larger leaves compared to the wild-type plants (Figure 6a). Additionally, the fresh weight of both wild-type and overexpressing plants was measured after mannitol stress treatment at concentrations of 0 mM, 200 mM, 250 mM, and 300 mM (Figure 6c). There was not much difference in fresh weight between 10 seedlings of WT and overexpression strains when treated with 0 mM mannitol; at 200 mM, the fresh weight of WT (6 mg) was significantly lower than that of the overexpression strain (about 7 mg). With the increase in stress concentration, the fresh weight of WT seedlings gradually decreased, while the overexpression strain maintained a high fresh weight. Under 300 mM mannitol stress, the fresh weight of WT was only about 3.4 mg, while the # 2 strain reached as high as 5.6 mg. This finding suggests that, compared with wild-type tobacco, the *LcWRKY40*-overexpressing tobacco exhibited a certain degree of drought resistance during the seedling stage.

#### 2.5.3. Alkaline Salt Tolerance Growth Characteristics of *LcWRKY40*-Overexpressing Tobacco

**Figure 7:**
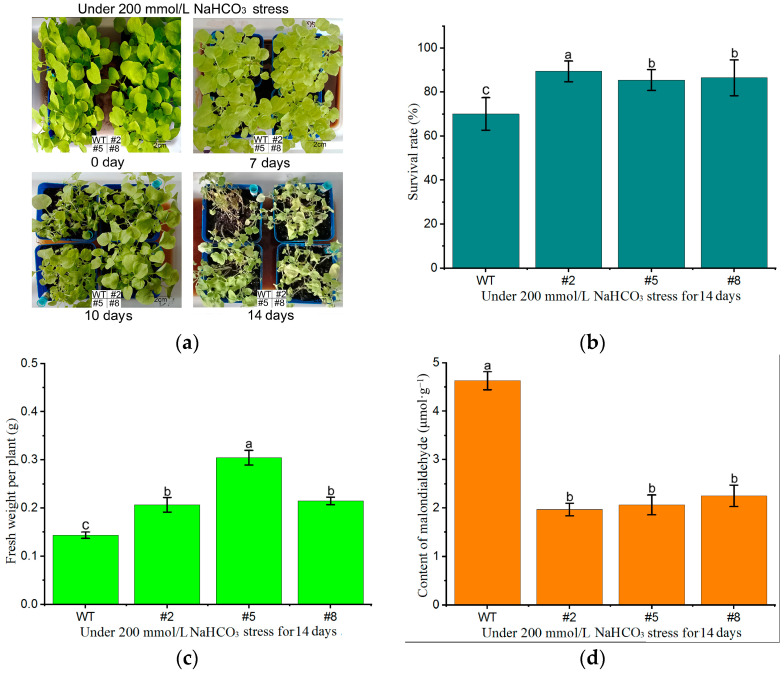
Characterization of the growth of tobacco plants expressing the *LcWRKY40* gene for alkaline salt tolerance: (**a**) phenotypes of plants treated with NaHCO_3_ root irrigation after treatment for 0, 7, 10, and 14 days; (**b**) survival rate of plants treated with NaHCO_3_ root irrigation after 14 days; (**c**) fresh weight of a single plant; (**d**) malondialdehyde content. Lowercase letters a, b, and c indicate significant differences between the overexpression strains and wild-type plants (*p* < 0.05).

The overexpression lines (T3-#2, #5, and #8) exhibited inhibited growth and leaf chlorosis upon root irrigation with 200 mM NaHCO_3_ for 7 days at the three-leaf stage. At this point (as shown in Figure 7a), the differences between the overexpression lines and the WT plants were not significant. Further observation of the treated plants revealed severe wilting and increased mortality after 10 days of treatment. During this period, a noticeable difference in tolerance to alkaline stress was observed between the overexpression lines and the WT. After 14 days of treatment, the survival rate of the overexpression lines was consistently greater than that of the WT, reaching more than 85% (Figure 7b). The T3-#5-overexpression line had the highest fresh weight (0.3046 g), followed by the T3-#2 and T3-#8 lines, while the WT exhibited the lowest fresh weight (0.1439 g) (Figure 7c). Moreover, the evaluation of malondialdehyde (MDA) content after stress exposure indicated that the WT plants had the highest MDA content, whereas the overexpression lines had significantly lower MDA levels than did the WT plants (Figure 7d). This finding suggests that the transgenic overexpression plants suffered less oxidative damage than the WT plants under NaHCO_3_-induced alkaline salt stress, demonstrating their resilience to this stress condition.

**Figure 8:**
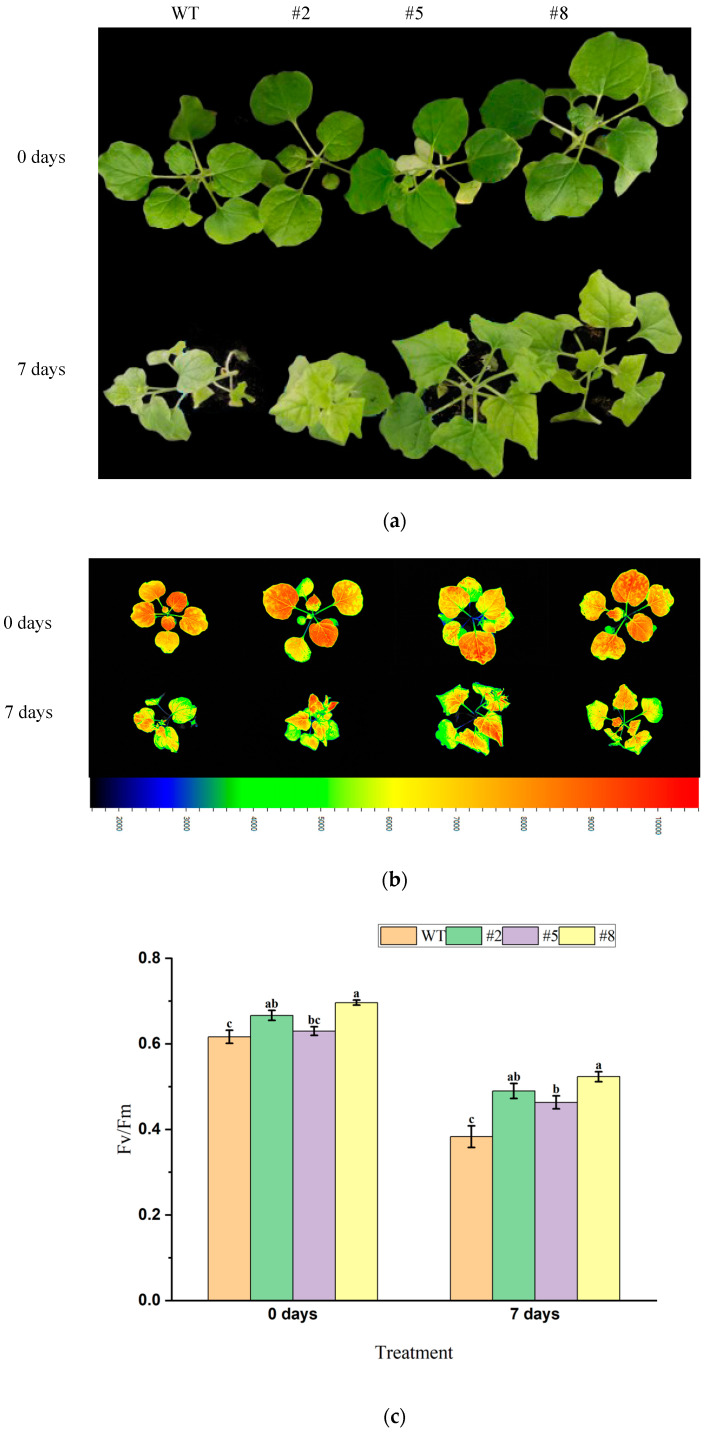
Salt tolerance of mature transgenic tobacco overexpressing *LcWRKY40*: (**a**) phenotypes of 200 mM NaHCO3 root irrigation treatment for 7 days; (**b**) chlorophyll fluorescence parameter images; (**c**) comparison of Fv/Fm values between wild-type tobacco and transgenic tobacco overexpressing *LcWRKY40*. a, b, and c indicate significant differences between transgenic lines and wild-type plants (at the *p* < 0.05 level).

Transgenic tobacco lines with seven leaves and one shoot (T3 generation) overexpressing *LcWRKY40* were subjected to 200 mM NaHCO_3_ root irrigation treatment for 7 days (Figure 8a). Chlorophyll fluorescence imaging under optimal/maximal photochemical efficiency of PSⅡin the dark (Fv/Fm) mode was obtained using the FluorCam open system (Figure 8b). On day 0 of the treatment, both the wild-type and transgenic lines exhibited similar growth and were in good condition. By day 7, the plants displayed slight chlorosis and wilting, with mild lodging, which was more pronounced in the wild-type plants (Figure 8a). Measurements using the chlorophyll fluorescence imaging system revealed that the maximum photochemical efficiency (Fv/Fm) ratio of the transgenic lines was better than that of the wild-type lines (Figure 8c). These results indicate that under 200 mM NaHCO_3_ root irrigation treatment, the PSII reaction center in wild-type lines suffered greater damage. The *LcWRKY40* gene appears to mitigate photodamage, enhancing the plants’ resistance to alkaline salt stress.

**Figure 9:**
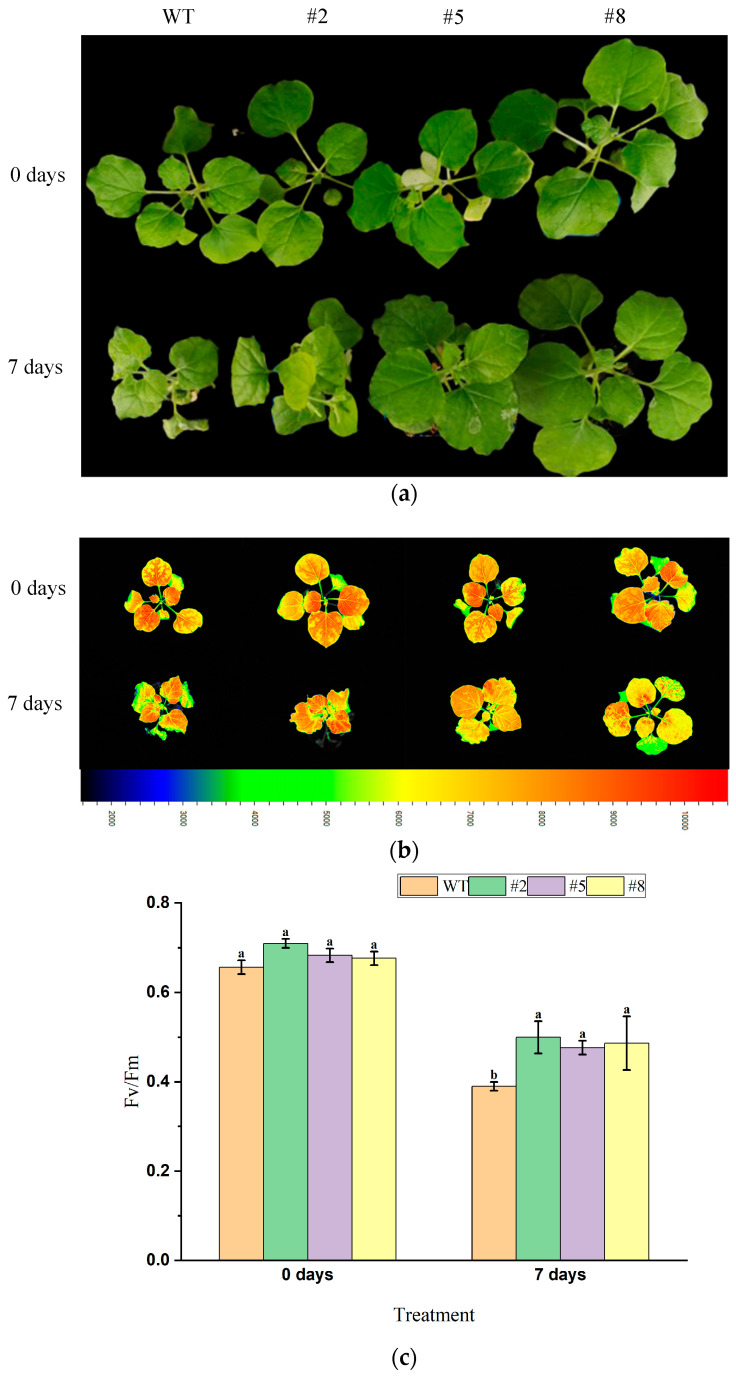
Alkaline salt tolerance of mature transgenic tobacco overexpressing *LcWRKY40*: (**a**) phenotypes of 300 mM NaCl root irrigation treatment for 7 days; (**b**) chlorophyll fluorescence parameter images; (**c**) comparison of Fv/Fm values between wild-type tobacco and transgenic tobacco overexpressing *LcWRKY40*. a, b indicate significant differences between transgenic lines and wild-type plants (at the *p* < 0.05 level).

Similarly, transgenic tobacco lines at the seven leaves and one shoot stage (T3 generation) overexpressing *LcWRKY40* were subjected to 300 mM NaCl root irrigation treatment for 7 days (Figure 9a). Chlorophyll fluorescence imaging under Fv/Fm mode was obtained using the FluorCam open system (Figure 9b). Under NaCl stress, wild-type lines exhibited significant wilting and growth restriction, while the transgenic lines showed a significantly better phenotype. Analysis of the Fv/Fm ratio revealed that the transgenic lines had a higher Fv/Fm ratio than the wild-type lines (Figure 9c), indicating that the *LcWRKY40* gene can reduce photodamage under NaCl stress, thereby improving the plants’ salt tolerance.

**Figure 10:**
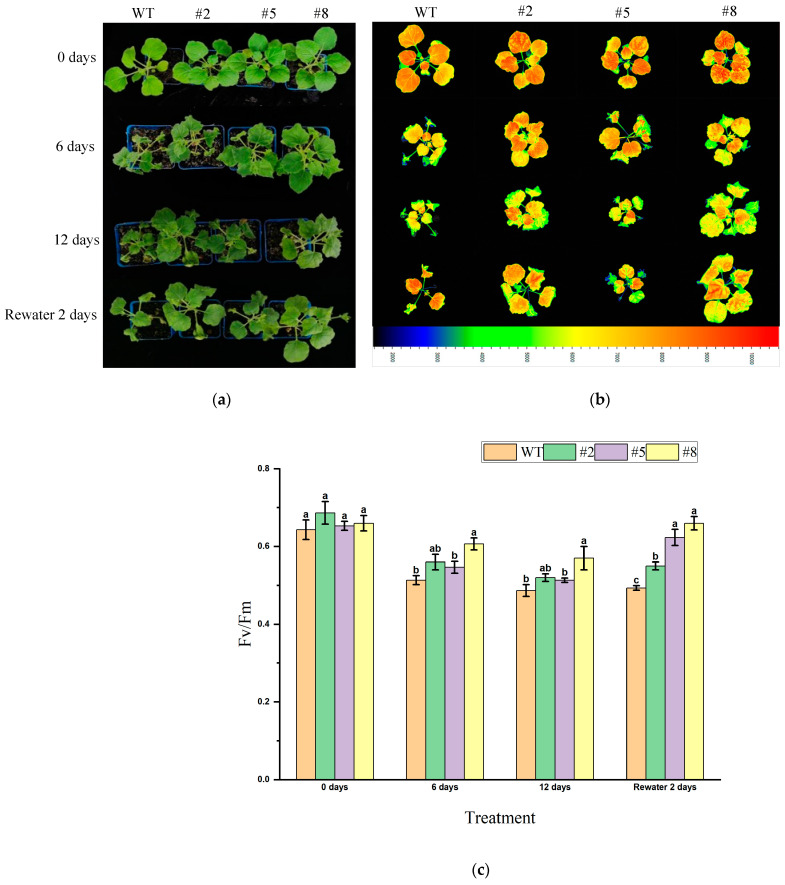
Drought tolerance of mature transgenic tobacco overexpressing *LcWRKY40*: (**a**) phenotypes at 0, 6, and 12 days of natural drought, and after 2 days of rehydration; (**b**) chlorophyll fluorescence parameter images; (**c**) comparison of Fv/Fm values between wild-type tobacco and transgenic tobacco overexpressing *LcWRKY40*. a, b indicate significant differences between transgenic lines and wild-type plants (at the *p* < 0.05 level).

Additionally, transgenic tobacco lines at the vegetative stage (T3 generation) overexpressing *LcWRKY40* were subjected to natural drought for 12 days with the wild-type strain, followed by rehydration for 2 days. Under natural drought conditions, all plants exhibited wilting, with the wild-type lines showing more pronounced symptoms. After 2 days of rehydration, the transgenic lines gradually recovered (Figure 10a). Chlorophyll fluorescence imaging under Fv/Fm mode was obtained using the FluorCam open system. As the drought duration increased, the F0 value of the wild-type lines showed a more noticeable increasing trend, and the Fv/Fm ratio decreased more significantly compared to the transgenic lines (Figure 10b). By comparing the Fv/Fm ratios on days 0, 6, 12 of drought, and after 2 days of rehydration (Figure 10c), it was found that the Fv/Fm value of the wild-type lines significantly decreased under natural drought conditions. After rehydration, the Fv/Fm value of the transgenic lines increased significantly, and their Fv/Fm value remained higher than that of the wild-type lines throughout the drought period. These results suggest that the *LcWRKY40* gene can reduce photodamage during drought stress, enhancing the plants’ drought resistance.

## 3. Discussion

**Figure 11:**
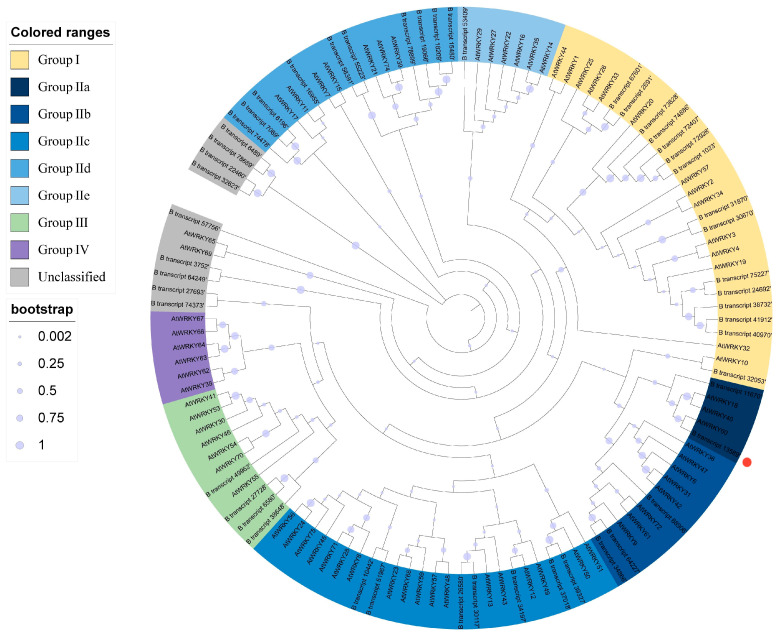
Phylogenetic tree of WRKY transcription factor sequences in *Leymus chinensis* under NaHCO_3_ stress and of the *Arabidopsis* WRKY transcription factor superfamily. The red dots represent *LcWRKY40*. Different transcription factors are grouped in different colors.

The ecological challenges posed by drought and soda saline-alkali soils in the Songnen Plain severely hinder grassland ecosystem development and forage growth, resulting in reduced grass production both in quantity and quality. In response to these environmental stressors, this study investigates the potential of the *LcWRKY40* gene in enhancing stress tolerance in *Leymus chinensis*. *L. chinensis* is a dominant species in the saline-alkali meadows and grasslands of the Songnen Plain, and its resilience is inseparable from its genetic evolution toward nonbiological stress. The initial exploration of *L. chinensis* plants under salt (NaCl) and alkali (NaHCO_3_) conditions using 454-FLX sequencing of gene fragments with an average length of 490 bp revealed 36,497 upregulated genes and 18,218 differentially expressed genes (DEGs) under these conditions [18]. While this initial analysis yielded important DEGs in response to salt–alkali stress, the sequences were incomplete, necessitating further analysis using full-length transcriptome sequencing. To address this limitation, our research team treated *L. chinensis* seedlings with alkaline salt (NaHCO_3_) and utilized PacBio instrumentation for full-length transcriptome sequencing, obtaining 160,559 full-length nonchimeric sequences. Sequence comparison of the transcriptome data with the reported *Arabidopsis thaliana* WRKY transcription factor superfamily revealed that B transcript 13,589 has a closer evolutionary relationship with *AtWRKY40* [19] and was thus named *LcWRKR40* (Figure 11). This was subsequently cloned, and its functional role in drought and saline-alkali tolerance was investigated.

Given the importance and drought resistance of *L*. *chinensis* in grassland restoration in saline alkali land, this study analyzed the expression patterns of the *LcWRKY40* gene under drought and alkaline salt induction. In *L. chinensis* seedlings, *LcWRKY40* expression increased in response to PEG treatment, peaking at 9.03 times the control level after 12 h, then gradually decreased. Under NaHCO_3_-induced stress, *LcWRKY40* expression in leaves and roots surged, reaching a peak of 26.19 times the control level at 48 h (Figure 2). In the context of exploring stress resistance genes and their underlying mechanisms [20], it was found that the *LcWRKY40* gene responded to drought and alkaline salt stresses, suggesting its involvement in regulating these stressors. It was localized to the cell nucleus (Figure 3) and is consistent with other WRKY transcription factors, such as *GhWRKY40* [21]. *PbWRKY40* has been shown to enhance salt tolerance by modulating downstream stress response genes in pears, and we have also conducted a series of validation experiments on the *LcWRKY40* gene [22]. Analysis of yeast expressing the *LcWRKY40* gene driven by the GAL4 promoter under drought and salt–alkali stress revealed significant differences when a 10^−4^ yeast dilution was plated on 2 M sorbitol and 30 mM NaHCO_3_ plates compared to the control (Figure 4). The increased expression of the *LcWRKY40* gene enhanced yeast clone growth under drought and alkaline salt stresses, leading to increased tolerance to stressors. This increase in stress tolerance in yeast resembles the strong stress resistance observed in yeast cells overexpressing the LSD1-type zinc finger protein *OsLOL5* [23]. A comparative analysis of drought resistance in tobacco plants overexpressing *LcWRKY40* under 300 mM mannitol stress revealed a significant increase in the fresh weight of the plants in the late germination stage compared to that of the WT plants (Figure 6). NaHCO_3_ root irrigation inhibited tobacco plant growth, causing wilting, decreased fresh weight, and plant death. However, the overexpression lines showed distinct differences in growth resilience compared to WT, with higher survival rates and significantly lower levels of the oxidative damage indicator malondialdehyde (MDA) compared to WT (Figure 7). These findings indicate that the *LcWRKY40* gene, under the control of the 35S promoter, possesses regulatory characteristics that can enhance tobacco drought and alkaline salt resistance. To evaluate the impact of NaCl stress on photosystem II efficiency, Fv/Fm values were measured using the FluorCam chlorophyll fluorescence imaging system. The results showed that transgenic lines maintained significantly higher Fv/Fm values compared to wild-type plants (Figure 8, Figure 9 and Figure 10).

This study exemplifies the role of WRKY family members in the biological processes of tolerance to various stressors [24]. In wheat, *TaWRKY17* enhances salt tolerance by regulating ABA/ROS-related genes and stress-responsive genes, consequently augmenting antioxidative stress resistance [25]. The overexpression of *AnWRKY29*, obtained from the shrub Nantucket, enhances the antioxidant and osmoregulatory capacities of transgenic plants; thus, *AnWRKY29* plays an important role in the drought tolerance pathway in plants [26]. Whether *LcWRKY40* can also modulate ROS synthesis and enhance the antioxidant stress capacity of plants needs further investigation. In addition, the WRKY transcription factor has been found to enhance plant resistance to heavy metals such as iron: *MxWRKY53* [27]. Whether *LcWRKY40* can also enhance plant growth under heavy metal stress needs to be further studied. The response of the *LcWRKY40* gene to drought and alkaline salt (NaHCO_3_) stress induction involves a regulatory network of factors associated with drought resistance and saline-alkaline stress. These findings offer valuable insights for crop breeding and genetic engineering aimed at developing drought- and salt–alkali-tolerant crops. By utilizing *LcWRKY40,* future efforts could focus on improving the resilience of economically important species in arid and saline environments.

## 4. Materials and Methods

### 4.1. Plant Material

Seeds of *L. chinensis* were collected from the salt–alkaline grassland of the Andal Experimental Station, a key laboratory for vegetation restoration and reconstruction in the northeastern region.

Transgenic *Nicotiana benthamiana* T3 generation seeds overexpressing the *LcWRKY40* gene were genetically transformed and preserved through propagation in our laboratory.

Protoplast cells of Longjing 11 rice were provided by the research team of Qingyun Bu at the Northeast Institute of Geography, Agriculture and Ecology.

### 4.2. Strains and Vectors

The yeast (*Saccharomyces cerevisiae*) strain INVScI was purchased from Invitrogen (Shanghai, China) (Item number: C81000), and it was stored at −80 °C fridge in the Key Laboratory of Salt alkali Vegetation Restoration and Reconstruction of the Ministry of Education in Northeast China (Northeast Forestry University). The yeast expression vector pYES2 and the pBI121::GFP plant expression vector for plant fluorescence expression were stored as above. The plant expression vectors pBI121::*LcWRKY40::GFP* and pCXSN::*LcWRKY40* were constructed by our laboratory.

### 4.3. Drugs and Reagents

RNA extraction kits were obtained from ComWin Biotech Co., Ltd. (Beijing, China). Reverse transcription kits were procured from Beijing Jialan Biotech Co., Ltd. (Beijing, China). The yeast plasmid extraction kit was purchased from Omega Bio-tek, Inc. (Norcross, GA, USA). Yeast nitrogen base powder (Y0626) and uracil DP supplement powder (Y1501) were obtained from SIGMA. NaHCO_3_ (NaHCO_3_ content not less than 99.5%), PEG6000 (the clarity test is less than or equal to 6) were sourced from Yongda chemical reagent Co., Ltd. (Tianjin, China).

### 4.4. Methods

#### 4.4.1. Cloning of *LcWRKY40* Gene

An analysis of 51 *WRKY* gene fragments annotated from the transcriptome sequencing of *L. chinensis* under NaHCO_3_ stress identified the alkaline salt-induced gene *LcWRKY40* (QIG37591), with NCBI accession number MN187915. Primers were designed using its CDS sequence in Primer5.0 software: *LcWRKY40*-F1: GCTTGGTGTTTGAGAGACGA and *LcWRKY40*-R1: TGATGTGGCTGCTCCTGTG. The target gene was amplified using RT-PCR technology.

#### 4.4.2. Expression Profiling of the *LcWRKY40* Gene in the Leaves and Roots of *Leymus Chinensis* under PEG6000 and NaHCO_3_ Stresses

To investigate the expression of the *LcWRKY40* gene (QIG37591) in *L. chinensis* seedlings under drought and alkaline salt stresses, this study used PEG6000 to simulate drought stress and NaHCO_3_ treatment to simulate alkaline salt stress. Seeds were sterilized and sown in a 1:2 mixture of vermiculite and perlite. The seedlings were subsequently grown in 1/2 MS nutrient solution until they reached the four-leaf stage with one shoot. At this stage, the plants were separately treated with 20% PEG6000 or 40 mmol/L NaHCO_3_. Samples were collected from roots and leaves at 0, 6, 12, 24, and 48 h post treatment. Total RNA was isolated and purified using the ComWin RNA Extraction Kit, followed by cDNA synthesis from 1 μg of total RNA as a template using the Beijing Jialan reverse transcription kit. The RT-qPCR method, as described by Danni Li et al., was used to analyze the temporal expression of genes after stress in various tissues and organs. The data were collected using the MxPro-Mx3000P system, and each experimental sample was analyzed in triplicate. Origin (2024b) software was used for data analysis and visualization.

#### 4.4.3. Subcellular Localization Analysis of the LcWRKY40 Protein

To predict the subcellular localization of the LcWRKY40 protein within the cell, the online platform https://wolfpsort.hgc.jp/ (accessed on 6 June 2024) was utilized. Subsequently, transient transformation of the rice protoplasts was performed using the recombinant expression vector pBI121*::LcWRKY40::GFP* and the control vector pBI121*::GFP.* This procedure was used to examine the subcellular localization of the LcWRKY40 protein. Rice protoplast extraction and transformation of the recombinant plasmids were carried out following the methods described by Bart R [28] and Zhang Y [29].

Fluorescence was observed in the transformed rice protoplast cells using a laser confocal microscope (Olympus, Tokyo, Japan) 14–16 h post transformation. The subcellular functional localization of the LcWRKY40 protein was determined by observing the merged GFP fluorescence signal.

#### 4.4.4. Analysis of Sensitivity to Mannitol and Saline-Alkaline Stress in Yeast Overexpressing Recombinant pYES2::*LcWRKY40*

This study aimed to explore the drought and salt–alkali resistance characteristics of the *LcWRKY40* gene in yeast cells. To construct the yeast overexpression vector pYES2*::LcWRKY40*, the plasmid pBI121*::LcWRKY40::GFP* was diluted 200-fold, and DNA fragments were amplified by PCR using specific primers (*LcWRKY40*-F3: 5′-GAATTCATGGATCCATGGGTCAG-3′; *LcWRKY40*-R3: 5′-CTCGAGTTAATTGATGTCCCTGG-3′). Subsequently, the DNA fragments were digested and ligated into the pYES2 vector, which contained the same restriction enzyme sites. The resulting pYES2*::LcWRKY40* plasmid was then heat-transformed into the yeast strain INVScI. The transformed yeast strains harboring the verified pYES2*::LcWRKY40* gene and those transformed with pYES2 were cultured on SD/-Ura plates. Single clones were picked and cultured in YPD liquid media at 30 °C for 1–2 days. The initial concentration was adjusted to OD_λ600_ = 0.6. Then, the cultures were diluted with sterile water to obtain five different concentrations (10^−1^, 10^−2^, 10^−3^, 10^−4^, and 10^−5^), of which 3 µL of each yeast suspension was spotted onto YPG solid media supplemented with various concentrations of sorbitol (sorbitol: 1, 2 M), NaHCO_3_ (NaHCO_3_: 10, 20, 30 mM), or NaCl (NaCl: 200, 300, 500 mM). YPD solid culture medium without any stress was used as the control group, and sampling was performed in the same manner. The cultures were incubated at a constant temperature of 30 °C, and after 2–3 days, the differences in colony growth were observed and recorded.

#### 4.4.5. Overexpression in Tobacco: Resistance and Phenotypic Analysis

- Obtaining Transgenic Tobacco Overexpressing the *LcWRKY40* Gene:

The constructed recombinant plasmid pCXSN::*LcWRKY40* was transformed into Agrobacterium tumefaciens strain GV3101 to obtain the correct Agrobacterium suspension. The transgenic tobacco plants were prepared using Agrobacterium-mediated transformation. Wild-type tobacco leaves were immersed in the bacterial suspension for 5 min and then placed on tobacco coculture medium (1/2 MS + As) and incubated in the dark at 25 °C for 3 days. After that, the leaves were transferred to the selection medium (1/2 MS + 0.5 mg/L 6BA + 0.1 mg/L NAA + 50 mg/L Hyg + 200 mg/L Carb) and incubated at 25 °C. Once the healing tissue had developed new shoots, they were transferred to the subculture medium (1/2 MS + 50 mg/L Hyg + 200 mg/L Carb) and cultivated until they became mature plants.

- Molecular identification of the overexpressed *LcWRKY40* gene in tobacco:

We selected T3 generation plants #2, 3, 4, 5, 8, and wild-type (WT) seeds. We sterilized the seeds and sowed them on 1/2 MS medium containing 50 mg/L hygromycin (Hyg) for selection. We used 1/2 MS medium for control growth. After 25 days, we investigated the growth phenotypes. We transplanted Hyg-resistant seedlings and WT from the control plates into nutrient pots filled with a vermiculite and field soil mixture. When they reached the 5-leaf stage, we extracted the total RNA from the leaves and reverse-transcribed it into cDNA. Using specific primers (Actin-F: CATGCTATCCCTCGTCTCGACCT; Actin-R: CGCACTTCATGATGGAGTTGTAT; *LcWRKY40*-F2: TTGCCGTTCTTGAGTCGGAG; *LcWRKY40*-R2: GCGAAGGAGCACCTGAAGTA), we performed RT-PCR to detect and analyze the expression of the *LcWRKY40* gene in the corresponding lines of overexpressed tobacco. The pCXSN:: *LcWRKY40* plasmid was used as the positive control, and the WT cDNA was used as the negative control for agarose gel electrophoresis analysis.

- Analysis of the drought resistance characteristics of *LcWRKY40*-overexpressing tobacco plants:

Overexpressed T3 transgenic tobacco lines (#2, #5, and #8) were chosen to assess resistance to drought stress simulated by mannitol. We sowed the seeds of overexpression strains and WT separately in 1/2 MS medium supplemented with 200 mM, 250 mM, and 300 mM mannitol, and used 1/2 MS medium supplemented with 0 mM mannitol as a control. Germination rates were recorded from day 3 to day 10. Additionally, growth phenotypes after 20 days of stress treatment were observed, and comparisons were made regarding changes in plant fresh weight. The experiment was replicated three times.

- Analysis of alkaline salt tolerance growth characteristics in *LcWRKY40*-overexpressing tobacco plants:

Thirty uniform seeds of highly expressed lines (*Oe*-*LcWRKY40* T3-#2, #5, and #8) and WT strains were evenly sown in a 1:5 mixture of vermiculite and soil in nutrient pots. Under controlled conditions of 40 μmol/m^2^/s light quantum flux density, a temperature of 23 ± 1 °C, and humidity of 30 ± 2%, the plants were grown until the three-leaf stage. Root irrigation was conducted with 200 mM NaHCO_3_, ensuring that the nutrient pots were maintained in a soaked state by daily water supplementation to the base. Phenotypic observations were made after 7, 10, and 14 days of treatment. Survival rates were assessed on day 14, and measurements of individual plant fresh weight and malondialdehyde content were conducted.

To further assess the response of *LcWRKY40* transgenic plants to drought and salt–alkali stress in photosystem II, the FluorCam open chlorophyll fluorescence imaging system (Agilent, Santa Clara, CA, USA) was used to measure the Fv/Fm (maximum photochemical efficiency) of the nutritional WT and overexpressing tobacco lines under conditions of natural drought for 12 days followed by rehydration for 2 days, and stress conditions of 200 mM NaHCO_3_ and 300 mM NaCl for 7 days.

#### 4.4.6. Data Processing

The data were statistically analyzed using Excel (Office 2019), followed by variance analysis and significance testing performed using Duncan’s method in SPSS 24.0 software.

## Data Availability

The data that support the findings of this study are available from the corresponding author upon reasonable request.
